# Role of Statistical Random-Effects Linear Models in Personalized Medicine

**DOI:** 10.2174/1875692111201010022

**Published:** 2012-03

**Authors:** Francisco J Diaz, Hung-Wen Yeh, Jose de Leon

**Affiliations:** 1 Department of Biostatistics, The University of Kansas Medical Center, Mail Stop 1026, 3901 Rainbow Blvd., Kansas City, KS, 66160, USA; 2 University of Kentucky Mental Health Research Center at Eastern State Hospital, Lexington, KY, United States, 627 West Fourth St., Lexington, KY 40508, USA

**Keywords:** Chronic diseases, dosage individualization, drug mixed linear models, effect sizes, empirical Bayesian feedback, evidence farms, pharmacokinetic modeling, random-effects linear models.

## Abstract

Some empirical studies and recent developments in pharmacokinetic theory suggest that statistical random-effects linear models are valuable tools that allow describing simultaneously patient populations as a whole and patients as individuals. This remarkable characteristic indicates that these models may be useful in the development of personalized medicine, which aims at finding treatment regimes that are appropriate for particular patients, not just appropriate for the average patient. In fact, published developments show that random-effects linear models may provide a solid theoretical framework for drug dosage individualization in chronic diseases. In particular, individualized dosages computed with these models by means of an empirical Bayesian approach may produce better results than dosages computed with some methods routinely used in therapeutic drug monitoring. This is further supported by published empirical and theoretical findings that show that random effects linear models may provide accurate representations of phase III and IV steady-state pharmacokinetic data, and may be useful for dosage computations. These models have applications in the design of clinical algorithms for drug dosage individualization in chronic diseases; in the computation of dose correction factors; computation of the minimum number of blood samples from a patient that are necessary for calculating an optimal individualized drug dosage in therapeutic drug monitoring; measure of the clinical importance of clinical, demographic, environmental or genetic covariates; study of drug-drug interactions in clinical settings; the implementation of computational tools for web-site-based evidence farming; design of pharmacogenomic studies; and in the development of a pharmacological theory of dosage individualization.

## 1 INTRODUCTION

Traditionally, statistical nonlinear models derived from compartmental theory have been used to represent and analyze the data obtained in population pharmacokinetic studies; to investigate the effects of clinical, genetic, environmental or demographic covariates on important pharmacokinetic parameters such as drug clearance; and to delineate treatment regimes based on the obtained information. However, a growing body of evidence suggests that another family of statistical models, usually called random-effects linear models (or linear mixed models), may provide a solid conceptual framework and valuable tools for the development of personalized medicine. In this article, we review empirical and theoretical evidence that suggests that these models may be very useful for pharmacokinetic research and that, in some situations, working with these models may be more advantageous and produce more reliable results than working with nonlinear models. This article also describes proposed applications of random-effects linear models, and describes recent developments in pharmacological theory that suggest that random effects linear models may play a significant role in personalized medicine in the future.

Population pharmacokinetics, which has traditionally been used to investigate how clinical, genetic or demographic covariates interact with dosages, uses random-effects statistical models [1]. These models have traditionally been nonlinear, which is due to the fact that the structural parts of these models are solutions of differential equations that represent the human body as a set of anatomical or physiological compartments. This conceptualization of the human body has provided strong methodological tools for supporting pharmacokinetic research, although the resultant nonlinear models are sometimes difficult to implement in practice. 

The discussion in this paper aims at informing clinicians and pharmacologists about random-effects linear models as promising tools in personalized medicine. It will also serve as a tutorial to introduce some statistical concepts and terminology used by statisticians in the context of these models. This article focuses on the applications of random-effects linear models, with emphasis on applications to drug dosage individualization and personalized medicine. Applications of random-effects nonlinear models can be found, for instance, in Pillai *et al.* [1]. In this article, we highlight the advantages of linear over non-linear mixed models in the context of pharmacokinetic research and personalized medicine. This article highlights the applications of random-effects linear models to drug dosage individualization, to the computation of effect sizes for assessing the clinical importance of covariates on pharmacokinetic or pharmacodynamic responses, and to the study of drug-drug interactions. We also describe the potential of these models for implementing evidence farming, which are web sites assisting health care providers to tailor medical treatments [2, 3]; and the potential for identifying genetic variants relevant to pharmacokinetic or pharmacodynamic responses in large-scale pharmacogenomic studies. A summary of applications is given in Table **1**. 

## 2 WHY SHOULD WE USE RANDOM-EFFECTS MODELS IN PERSONALIZED MEDICINE?

The feature that makes random-effects (linear or non-linear) models so useful for personalized medicine is that a random coefficient can be viewed as a parameter that is a characteristic constant for a particular patient, but that varies across patients [4-6]. In this sense, the variability of a random coefficient is considered to be the result of real variation in biological and environmental factors, and not just a mathematical trick to handle the variability of patients’ pharmacological response. This suggests that random-effects statistical models, which are also called “mixed models” or “variance components models”, should be used in pharmacokinetics and personalized medicine because they allow consideration of a patient as an individual with unique characteristics, not just as a member of a population that has an average value to be understood. 

### 2.1 The Random Intercept Linear Model

Fig. (**1**) gives a description of some of the concepts related to random-effects linear models. To keep things simple, we will describe a simple version of a random-effects linear model, namely the random-intercept linear model, which has potential applications to drug dosage individualization. Diaz *et al.* [5, 6] proposed using a model of a pharmacokinetic or pharmacodynamic response from a particular patient that assumes that the response, the drug dosage, and the clinical, environmental, biological or demographic covariates of a particular patient are related through the following equation:

(1) logYD=α+βTX+d logD+ε.

Here, *Y_D_* is a stable pharmacokinetic or pharmacodynamic response, *D* is the particular drug dosage that produced this response, ***X*** is a vector of (demographic, clinical, genetic or environmental) covariates, ***β*** is a vector of unknown regression coefficients that need to be estimated by using a sample of patients, *d* is the regression coefficient for the natural log of dosage, which also needs to be estimated, and *α* is a characteristic constant of the individual. At the individual level, *α* is considered a constant number that characterizes the patient. However, at the patient population level, *α* is viewed as a random variable in the sense that *α* is a number that varies from patient to patient and its mean is equal to the population average of the characteristics. For this reason, *α* is called a random intercept. What makes this model useful for personalized medicine is that it includes a parameter *α* that identifies the patient. This parameter is usually estimated (or “predicted”) by combining information from the particular patient with information from the population of patients to which the particular patient belongs. The more information we have from the patient, the more accurate the estimation is. 

The population mean and variance of *α* are denoted by *µ_a_* and $\sigma_{\alpha }^{2}$, respectively. Also, ε is an intra-individual random error that is assumed to be statistically independent of α. The variance of α is viewed as a measure of the inter-patient variability across the entire population of patients, whereas the variance of ε as a measure of the intra-patient variability. Statisticians call ***β***, *d* and *µ_a_* the fixed effects of model (1); a reason for this terminology is that these numbers are considered to be population constants, that is, fixed numbers that do not vary from one patient to another. In this sense, ***β*** , *d* and *µ_a_* are viewed as numbers that represent the “average subject”. In a more flexible (but more complex) random-effects model, ***β*** may also be assumed random in the sense that the effects of covariates on the stable pharmacokinetic or pharmacodynamic response vary from patient to patient. Correspondingly, in this case the model is called a random-slope linear model (with or without a random intercept). See Fig. (**1**) (b) for a simple model with a random slope but a non-random intercept, and (c) for a model with both random intercept and random slope. For pedagogical reasons, this article focuses on model (1); the general situation in which ***β*** is considered random is studied in reference [6].

**Fig. (1):**
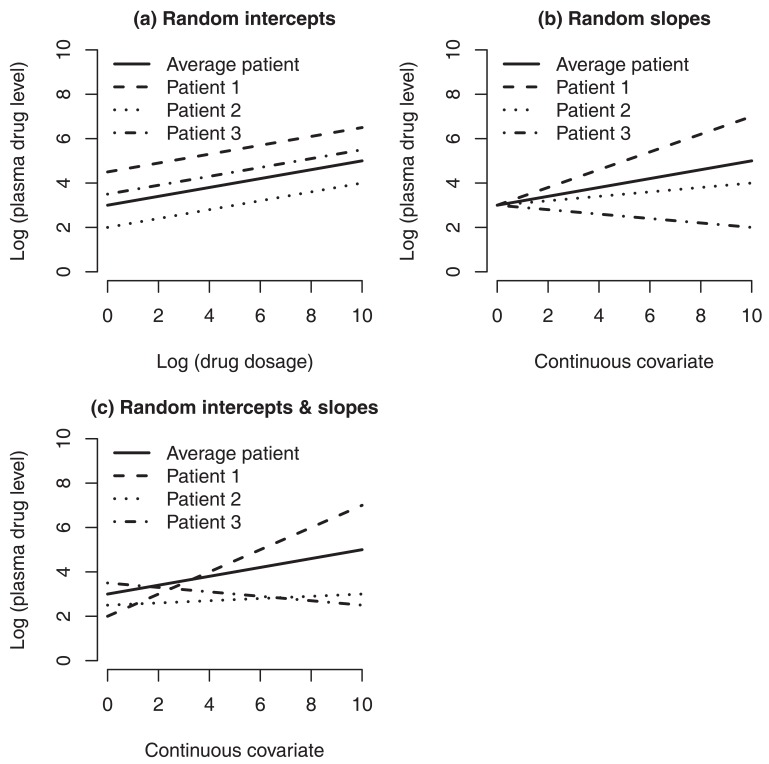
***Random-effects linear models do not only represent the average patient but also individual patients***. The simplest, but a very useful version, of these models is the random intercept linear model, illustrated in part a; in this part, patient 1 eliminates the drug from blood more slowly than patient 3, since drug levels are higher in patient 1 for any administered dosage. In part a, it is assumed that the patients have the same values in the clinical, demographic, biological and environmental covariates that affect drug levels. Part b illustrates a situation in which a covariate has random effects; the effect of the covariate on drug levels is stronger in patient 1 than in patient 2. Part c shows a situation in which a covariate has random effects and there is a random intercept. In parts b and c, it is assumed that the patients are under comparable dosages and are comparable in other covariates. It is usually hypothesized that most of the inter-patient variation caused by unexplained genetic variability is absorbed into the variability of the random intercept or slopes, and that the variability of the error term is never explained by genetic variation.

It must be noted that, to be able to estimate the parameters of a random-effects linear model, it is necessary to measure the pharmacological response *Y_D_* several times in several patients; that is, repeated measures must be obtained. The inter- and intra-patient variabilities cannot be separated otherwise. A reader familiar with linear regression but not expert in statistics may be tempted to consider equation (1) as just the classic linear regression model applied to the natural log of the pharmacological response *Y_D_*. This way of seeing equation (1) is not totally wrong, but is not exactly right. In fact, equation (1) describes only one patient, not the population of patients. A random intercept linear model is a set of many equations similar to equation (1), where two equations may possibly differ in the value of α, and the equation corresponding to one patient has a unique value of α (see Fig. **1a**). In other words, the population of patients is represented by a population of equations like that in (1). This allows incorporating patients’ idiosyncrasies and identities in theoretical pharmacological developments. In contrast, the classic linear regression model consists of only one equation whose intercept and all other regression coefficients are fixed population numbers that represent the entire population and allow modeling only average patients, not individual patients. If equation (1) represented a classic linear regression model, the error term ε would combine both inter- and intra-patient variation, hindering an effective isolation of patients’ individualities. 

### 2.2 Empirical Evidence Supporting the Use of Random-Effects Linear Models in Pharmacokinetic Analyses and Dosage Computations 

Empirical evidence in favor of using model (1) for pharmacokinetic analyses has been reported by a number of studies. Diaz *et al.* [5] applied this model in studying the effects of gender and smoking on the plasma concentrations of the antipsychotic clozapine, controlling for clozapine dosage. Diaz *et al.* [7] and Botts *et al*. [8] also used the model to investigate drug-drug interactions in clinical settings (see below). Specifically, interactions between clozapine and comedications were investigated by Diaz *et al.* [7], who found that smoking modified the size of the effect of the anticonvulsant valproic acid on plasma clozapine concentrations; and interactions between the antipsychotic olanzapine and comedications were studied by Botts *et al.* [8], who found that smoking modified the size of the effect of the anticonvulsant lamotrigine on the plasma concentrations of olanzapine. In the above studies, evidence was found that model (1) represented the pharmacokinetic data remarkably well [5, 7, 8]. 

In an independent work, Hu and Zhou [9] found a very close similarity between the (covariate-based) average dosage adjustment factors computed with traditional pharmacokinetic compartmental models and the factors computed with model (1) when the response *Y_D_* was a steady-state drug plasma concentration. They found this by examining 3 drugs (2 biologicals and 1 small molecule) administered to very large, multinational patient samples. This agreement between results of statistical analyses of steady-state pharmacokinetic data based on traditional pharmacokinetic nonlinear models and results based on model (1) was confirmed in another study by Hu *et al*. [10] who investigated a different, undisclosed drug. 

It is quite interesting to review how Hu and collaborators coincidentally found strong empirical evidence supporting model (1) [9, 10]. The original goal of these authors’ research was not to search for evidence supporting model (1). Instead, they used this model as the instrument of a “sensitivity analysis” to verify that their proposed approach to building compartmental nonlinear models was producing reasonable results. In their approach, population pharmacokinetic nonlinear models consisting of sums of exponentials were carefully built and fitted to steady-state drug plasma concentrations. One of the parameters of their nonlinear models was apparent clearance, which was treated as a random effect. Their main goal was to model apparent clearance as a function of clinical, demographic and biologic covariates. After selecting the covariates for their models, the sizes of the effects of the selected covariates on apparent clearance were estimated. Then they fitted model (1) in order to examine the sensitivity of the estimated effect sizes to substantial variations in the form of the model. Quite remarkably, model (1) produced essentially the same covariate effect sizes that their elaborate approach based on nonlinear models produced. The effect sizes produced by the two approaches were very similar to each other, and this was valid for each of the 3 drugs investigated by Hu and Zhou [9], and for the drug investigated by Hu *et al.* [10]. In fact, for each investigated drug, a combined plot of the covariate effect sizes and corresponding confidence intervals computed with Hu and collaborator’s approach was the mirror image of the analogous plot computed with the approach based on the random intercept linear model. (The reader is invited to compare Figs. (**1** and **3**) in Hu *et al.* [10]; the two figures are essentially identical.) Besides providing empirical support for model (1), Hu and collaborators’ findings suggest that steady-state pharmacokinetic data can be examined by using the less complicated (but still effective) random-effects linear models in place of the traditionally-used non linear models. 

A study on the clinical pharmacokinetics of risperidone suggests indirect empirical evidence that model (1) may accurately represent the relationship between steady-state total plasma risperidone concentrations, risperidone dosage and clinical and biological covariates [11]. Although this study did not use repeated measures of steady-state concentrations, it found that a classic linear regression model could be used to model the natural log of total risperidone concentration-to-dosage ratio, which is equivalent to using a linear regression model of the log of total risperidone concentrations in which the regression coefficient of the log of risperidone dosage is exactly 1. The study found that the log of total risperidone concentrations were significantly (and linearly) affected by the number of cytochrome P450 2D6 (CYP2D6) active alleles in the patient, the intake of comedications that induce the CYP3A enzyme system, the intake of comedications that inhibit the CYP system, and gender. This suggests that the random intercept linear model in (1) may represent very well risperidone concentrations from patients who have not been genotyped, because in that case the unmeasured CYP2D6 activity variation across patients would be captured into the variability of the random intercept. In other words, even if patients were not geno-typed, the variability of the random intercept of a random-effects linear model of total risperidone concentrations would reflect genetic variability (and the variability of some other variables not considered or measured). 

The idea that the variability of the random intercept in model (1) may be (at least in part) the result of the variability of alleles directly or indirectly involved in the body’s drug elimination, when the response *Y_D_* is a steady-state drug plasma concentration, is quite appealing. The essence of this idea, which is not new, underlies the published attempts by Kalow and coworkers [12-14] to measure the heritability of a pharmacological response by quantifying the inter- and intra-subject variabilities of the response. The essence of the idea can also be tracked back to the seminal work on statistical variance-components models by Henderson [15], who was motivated by the need to separate genetic and environmental influences in animal studies and laid out some of the mathematical foundations of mixed models. After fitting model (1) to clozapine data, Diaz *et al.* [5] found that the subjects' intercepts were significantly and positively correlated with an index of clozapine metabolic activity (the plasma clozapine/norclozapine concentration ratio); this supports the view that the variability of the random intercept may reflect (at least in part) biological differences across patients. Since it is not reasonable to explain the intra-patient variability of a pharmacokinetic or pharmacodynamic response by genetic variations, the genetic component of the variability of the response is usually considered to be completely incorporated in the inter-patient variability, which is the same as, and measured by, the variability of the random intercept in model (1).

### 2.3 Why is There a Close Agreement Between the Random Intercept Linear Model and Traditional Compartmental Nonlinear Models?

The remarkable agreement between covariate effect sizes and dosage correction factors provided by model (1) and those provided by traditional compartmental nonlinear models needs to be explained. As mentioned above, this agreement was found by Hu and Zhou [9] and Hu *et al.* [10] when working with steady-state pharmacokinetic data. It is possible to provide a reasonable explanation for this agreement, at least under the assumption of linear pharmacokinetics [i.e., when *d* = 1 in model (1)]. In this explanation, *Y_D_* is a steady-state drug plasma concentration in response to the drug dosage *D*. The drug concentration-to-dosage ratio is frequently considered a measure of the metabolic activity of an individual [16-18]. When *d* = 1, model (1) can be written in the following way, which emphasizes the role of this ratio in the model:

(2) $$\frac{Y_{D}}{D}=\exp\left(\alpha +B^{T}X+\varepsilon \right).$$

According to standard pharmacokinetic theory, the concentration-to-dosage ratio Y_D _/ D is essentially proportional to the multiplicative inverse of apparent clearance. By formula (2), the variability of essentially reflects the inter-patient variability of this ratio. Thus, exp(α) may be viewed as the portion of apparent clearance that is not explained by the covariates in ***X*** [9, 10]. 

Consistent with the above idea, Diaz *et al.* [5] proposed comparing the pharmacokinetic response of two individuals with comparable covariate values by using the quantity

(3) $$\gamma =\frac{\alpha -\mu_{\alpha }}{\sigma_{\alpha }},$$

Where *µ_a_* and *σ_a_* are the mean and standard deviation of the patients' random intercept α. If γ = 0, then the patient has a response that is similar to that of the average individual. If γ > 0, the patient eliminates the drug from blood more slowly than the average individual, and, if γ >, the patient eliminates it faster. According to this pharmacological interpretation of the random intercept, *γ* is a covariate adjusted proxy for apparent clearance and, when using trough steady-state drug plasma concentrations as the response *Y_D_*, model (1) quantifies the effects of covariates on apparent clearance. Since apparent clearance is the most important pharmacokinetic quantity to consider when designing a dosage regime for long-term drug administration [19], we can see now that model (1) has strong potential in the design of dosage regimes based on clinical, demographic, environmental and biological covariates and the unexplained inter-patient variability contained in α. In summary, model (1) is essentially a model of apparent clearance, which may explain why it provides characterizations of the effects of the above covariates on pharmacokinetic responses that are similar to the characterizations provided by compartmental nonlinear models in population studies.

## 3 RANDOM-EFFECTS LINEAR MODELS AND DRUG DOSAGE INDIVIDUALIZATION

Despite all determined attempts to find genetic variants whose identification in particular patients may allow tailoring particular pharmacologic treatments to those patients, the truth is that most genetic variants that have been discovered until now and that may serve that purpose explain only a small proportion of pharmacokinetic or pharmacodynamic responses. Moreover, much variability in pharmacokinetic and pharmacodynamic responses is not genetically determined and, assuming that behavioral factors such as treatment compliance can be reliably controlled during treatment, environmental factors also play a very important role [20, 21]. Thus, to model inter-patient variability with unequivocally identified genetic variants is a task that still remains elusive, and much more research is needed in order to find diagnostic methods based on genetic findings that allow individualizing treatments and drug dosages in a reliable way. 

However, personalized medicine may benefit not only from genetic research but also from population pharmacological studies that provide information about the way pharmacologic effects vary among different patients and about the factors that affect this variation. In fact, there is an emerging methodological area in personalized medicine whose aim is to develop statistical models that allow using the history of a chronically ill patient in order to define an optimal medical treatment for the patient. Before using one of these models in particular patients, the model needs to be calibrated first by using the histories of a representative sample of patients. These statistical models of chronic care, called dynamic treatment regimes, have recently been receiving some attention in the biostatistical literature and professional biostatistical meetings, probably because of the current renewed interest in personalized medicine, and because of some ground-breaking work that has allowed surmounting some of the mathematical and computational difficulties involved in this type of statistical modeling. These approaches, however, do not usually use mixed models and are usually grounded in (or justified by) nascent ideas from the machine learning community. A review of some of these approaches can be found in Chakraborty [22] and Henderson *et al.* [23]. However, the idea of dynamically (or “adaptively”) changing the treatment of a chronically ill patient by combining an estimated statistical population model with both historical and new information from the patient can be traced back to the work of Sheiner and collaborators in the 1970s and '80s, who proposed using nonlinear mixed models and empirical Bayesian approaches to achieve this purpose (see, *e.g*., [4] and the historical account in [1]). 

Diaz *et al*. [5] proposed a clinical algorithm for drug-dosage individualization based on random intercept linear model (1). This algorithm can be considered as a form of optimal dynamic treatment regime in which a parametric model of the pharmacokinetic or pharmacodynamic response is completely specified, namely model (1). The algorithm is not justified with machine learning concepts, but with the concepts of a solid general theory called Statistical Decision Theory, which prescribes widely accepted general principles for both the estimation of population parameters and the prediction of individual parameters in the presence of uncertainty [24]. The algorithm was extended by Diaz *et al.* [6] to the general situation in which some covariates have random effects. Here, we review the algorithm assuming that the population of patients satisfies model (1). The algorithm uses a procedure called Bayesian feedback which aims at improving the estimation of the patient’s intercept α by collecting more and more information from the patient. Solid theoretical arguments anchored in decision theory can be used to show that the clinical algorithm may provide better personalized dosages than those obtained through traditional therapeutic drug monitoring, provided model (1) describes adequately the population of patients. Some computer simulations support this claim as well [5, 6]. 

Diaz *et al.*’s algorithm [5, 6] is not a computer but a clinical algorithm. That is, the algorithm is a series of steps that the clinician should follow in order to obtain an optimal dosage for a particular patient from a population of patients with a chronic disease who satisfy model (1) of its generalizations. To explain the algorithm in the context of a desired pharmacokinetic response, some initial concepts are needed. Suppose we wish to produce a trough steady-state drug concentration lying within the therapeutic window (*l_1_*,*l_2_*), in which the drug will be effective and safe, where 0 < *l*_1 _ < *l*_2_. With this purpose, we search for an appropriate dosage *D*. Initially, the clinician will administer to the patient a dosage that is appropriate only for an average patient whose covariate values in ***X*** are similar to those of the patient. The main goal of the clinical algorithm is to improve the dosage *D* by improving the prediction of α (the term "prediction" is a technical word that can be understood as a synonym of "estimation", and by no means signifies that a future event is being forecasted). Before applying the algorithm to particular patients, the population parameters *µ_a_*, ***β***, *d*, $\sigma_{\alpha }^{2}$ and $\sigma_{\varepsilon }^{2}$ must be estimated by using a sample of patients, and the estimated model is considered as empirical prior information. In other words, the clinician individualizes the dosage of a particular patient by using empirical information that was obtained before applying the clinical algorithm (that is why this approach is called "empirical Bayesian"); this prior information is combined with the information from the patient in order to obtain an optimal dosage for the patient. Thus, two different but related concepts are involved in the drug dosage individualization procedure: the random effects linear model, and the clinical algorithm whose performance depends on the accuracy with which the model represents the patient population. 

Next we describe how the clinical algorithm is carried out. The only information that the clinician initially has from his/her patient is the values of the covariates in ***X***. In the first step of the algorithm, the clinician uses both the estimator of the population mean of α as a predictor of the patient’s α, and the patient’s covariate values. Thus, the initial dosage is

$$D_{1}={\left(\sqrt{l_{1}l_{2}}\exp\left(-\mu_{\alpha }-\beta^{T}X\right)\right)}^{\frac{1}{d}}$$

(Observe that the model parameters *µ_±_* ,² and *d* are used to compute the dosage, since these were estimated from the population before starting the dosage search for the individual patient; also, in the first step of the algorithm, the predictor of α is ${\hat{\alpha }}_{1}$ = $\mu_{\alpha }$ ). This initial dosage is administered appropriately to the patient and, once the steady-state is reached and just before a particular dose, a blood sample is taken from the patient and the trough drug plasma concentration $Y_{D_{1}}$ is measured. The clinician now has additional information from the patient that consists of both the initially administered dosage *D*_1_ and a measure of the produced patient's plasma concentration $Y_{D_{1}}$ . At the second step of the algorithm, this additional information is combined with the empirical prior information (the estimated model) in order to recompute the dosage. This combination of information allows computing a refined, better predictor of α, which allows recomputing a “more personalized” dosage. The formula used to combine this information is usually called “the empirical Bayes estimator of α” (also called the BLUP, best linear unbiased predictor of α), although some statisticians prefer using the term “empirical predictor” instead of “estimator”. The empirical Bayes estimator obtained at the second step, denoted ${\hat{\alpha }}_{2}$ is used to recompute the new dosage by using the formula

(4) $$D_{2}={\left(\sqrt{1_{1}1_{2}}\exp\left({-\hat{\alpha }}_{2}-\beta^{T}X\right)\right)}^{\frac{1}{d}}.$$

This new dosage is administered to the patient and, once the steady-state is reached, a new drug plasma concentration is obtained from the patient, say *Y_D2_*. Now the clinician has more information from the patient, namely the previously administered dosages *D_1_* and *D_2_* and the obtained plasma concentrations $Y_{D_{1}}$ and $Y_{D_{2}}$ , which are again combined with the empirical prior information to obtain a new, more precise empirical Bayes prediction of α, say ${\hat{\alpha }}_{3}$. The dosage is recomputed by using ${\hat{\alpha }}_{3}$ in place of ${\hat{\alpha }}_{2}$ in formula (4), and so on. 

In summary, if a patient’s covariate values is the only information from the patient that is initially available, an optimal rule for drug dosage individualization prescribes initially administering a dosage that is optimal only for the average individual, because the α of the patient is initially estimated by the population average of α. Then, the information provided by the patient afterwards should be used to update this rough estimator of α. This update is performed by combining this information with population information, and so on. This approach is usually called empirical Bayesian feedback because the information from the patient is combined with empirical prior information in order to improve our knowledge about the patient. Diaz *et al.* [6] also discuss how to use the algorithm when the clinician’s initial knowledge about the patient includes some of the patient’s responses to previously administered dosages in addition to knowledge about the patient’s covariate values. 

Diaz *et al.* [5, 6] demonstrated through theoretical arguments and simulations that if model (1) is an adequate description of the patients in the population, then the above clinical algorithm is optimal in the sense that, among many other possible drug dosage individualization algorithms, including those traditionally used in therapeutic drug monitoring (TDM), the above algorithm is the one that has the highest probability of making the patient’s plasma concentration reach the therapeutic window. In equivalent words, Diaz *et al.*’s algorithm minimizes the quantity 1 – P (*l*_1_ < *Y_Di_* < *l*_2_) at the *i* -th algorithm step; this quantity is called “the Bayes risk”. This optimality property of Diaz *et al.*’s algorithm is very appealing because, with other things considered, minimizing the probability that the pharmacokinetic response does not reach the therapeutic window is precisely what clinicians want for their patients. 

### 3.1 What is an Optimal Personalized Drug Dosage?

One important question that arises when applying Diaz *et al.*'s algorithm is when to stop the algorithm. Obviously, the clinician must stop the dosage search, at least temporarily, when an optimal dosage is achieved. Another important question is how to know that a particular drug dosage individualization procedure produces an optimal dosage or, at least, a dosage that is better than the dosages produced by other procedures. But, what is an optimal personalized dosage? It is clear that, in order to appropriately develop personalized medicine theory and practice, a precise definition of the term "optimal individualized drug dosage" needs to be provided. To assess the performance of their clinical algorithm, Diaz *et al.* [5, 6] proposed a definition of optimal dosage, or, more precisely, a definition of an *ω* -optimum dosage (read "omega optimum"). A dosage *D* is called *ω* -optimum for a patient with pharmacokinetic index *γ* if, after administering this dosage to the patient, the probability that the patient reaches the therapeutic window is close to the maximum probability that can be attained for that particular patient; more precisely, a dosage is *ω* - optimum if a fraction *ω* of the maximum attainable probability can be obtained with that dosage, where *ω* is a fixed fraction close to 1. [Recall that γ is defined in formula (3).] In general, the maximum attainable probability that a particular patient reaches the therapeutic window is never 1, and depends on both the ratio $\frac{1_{2}}{1_{1}}$ , a number that is usually called the therapeutic index, and the variance $\sigma_{\varepsilon }^{2}$ of the error ε. Unless the therapeutic window is too wide or the variance of the error is too small (something that sometimes is not obtainable in the real world), it is impossible for the clinician to compute a dosage that has 100% probability of producing a response within the therapeutic window. However, as shown by Diaz *et al.* [5, 6], it is possible that, after collecting enough information from the patient, the clinician computes a dosage that has a probability that is close to the theoretically maximum probability, provided that precise information about the patient population is previously obtained through a random-effects linear model and the above dosage-computation approach is used. For many drugs designed to treat chronic diseases, no drug dosage is 100% effective or non-toxic. However, random-effects linear models provide us with tools to deal with the real world in a rational and optimal way or, shall we say, up to the maximum effectiveness or non-toxicity that the real world allows us to have. 

One advantage of the above theoretical developments based on random-effects linear models is that important questions concerning personalized medicine may be answered. For instance, how much clinical information do we need from a particular patient in order to compute an optimal dosage for the patient? In particular, in a TDM setting, how many blood samples must be taken from the patient to ensure that an optimal dosage is computed? Random effects linear models provide a theoretical setting to answer questions like this. In particular, Diaz *et al.* [5] proved a theorem that provides the minimum number of blood samples that are necessary to obtain an ω-optimum dosage for a high number of individuals in the patient population. For instance, for the antipsychotic clozapine, computations show that only 3 or 4 blood samples from a patient may be sufficient to compute an optimal personalized dosage for the patient, at least with our current state of knowledge about this antipsychotic, and provided the above optimal individualization procedure is implemented [5, 6]. The point here is that a precise definition of optimal individualized dosage allowed answering the question of which algorithm step provides the clinician with an optimal dosage. 

Model (1) assumes that the covariates have fixed effects on the pharmacological or pharmacodynamic response *Y_D_*; that is, it assumes that covariate effects (the numbers in vector ***β***) are the same for all patients. However, in some situations, it may be more realistic to assume that the effect of a covariate may vary from patient to patient. For instance, Diaz *et al.* [6] found that smoking may have stronger effects on plasma clozapine concentrations in some patients than others. In this sense, smoking is said to be *a covariate with random effects*. This observation highlights the fact that in some situations unexplained individuality may be the result of not only unknown factors shaping the biology of individuals (which are partly represented in the model in the form of a random intercept), but also of unknown or unmeasured interacting factors that modify the effects of measured factors. As mentioned above, model (1) is the simplest random-effects linear model of a log-transformed response that can be built with pharmacological data. Diaz *et al.* [6] generalized the model in Diaz *et al.* [5] to situations in which some covariates have random effects, and described how to use the clinical algorithm in these situations. 

### 3.2 A Comparison with Traditional Therapeutic Drug Monitoring

By using simulations and arguments grounded in statistical decision theory, Diaz *et al.* [6] showed that Diaz *et al.* [5]’s algorithm may produce better personalized dosages than a method traditionally and very frequently used in TDM. The traditional method prescribes that, in order to improve a dosage by using a previously obtained drug level, the new dosage must be computed with the formula

$$\mathrm{adjusted dosage}=\frac{\mathrm{Previous dosage}}{\mathrm{Measured drug level}}C_{0},$$

where *C*_**0**_ is a target drug steady-state trough concentration. The above formula, which is usually justified with the theory of compartmental models and advocated in many pharmacological textbooks, produces overly suboptimal dosages in the sense that, even after taking a large number of blood samples from a patient, the adjusted dosage will never make the patient reach the therapeutic window with a probability as high as that produced by Diaz *et al.*'s algorithm. Given that there is strong empirical evidence suggesting that some drugs may be accurately modeled with random-effects linear models, this suggests that some current clinical approaches to drug dosage individualization used in TDM should be revised. 

## 4 ASSESSMENT OF THE CLINICAL IMPORTANCE OF COVARIATES

One advantage of using linear models is that their regression coefficients can be used to easily assess the clinical importance of clinical, genetic, environmental or demographic covariates to the variations of the pharmacokinetic or pharmacodynamic responses, and can be used to compute dose correction factors that account for the presence of drug-drug interactions [7, 8]. In fact, these measures of clinical importance, called "effect sizes", and the drug correction factors may even be easier to interpret and understand by practicing clinicians than pharmacokinetic quantities such as area under the curve or maximum plasma concentration [25]. 

When the dependent variable in a linear regression model is the log of a response, the importance of a covariate can be assessed by using effect sizes based on relative percentiles as explained below. The methodology for interpreting regression coefficients based on relative percentiles has been used not only in the context of random effects linear models [5, 7, 8] but also in classic linear regression models [11, 26]. Unfortunately, textbooks on linear regression models do not describe a way of interpreting regression coefficients when the dependent variable of the model is the log of a response. These textbooks usually teach that the regression coefficient of an independent variable in a linear regression model measures the average change in the dependent variable for each one-unit change in the independent variable. However, in contrast with the interpretation based on relative percentiles, this interpretation is not useful when the dependent variable is the log of a pharmacokinetic or pharmacodynamic response, because a pharmacologist is interested in understanding the effects of covariates on the response, not on the log-transformed response. 

The concept of relative percentile is very simple [27]. Suppose log(*W*_1_) and log(*W*_2_) are normal random variables, both with the same variance. Let 0 < *p* < 1; if *ω_i_*(*p*) is the *p* x 100% percentile of the response *W_i_*, *i* = 1,2, then the ratio of percentiles $\frac{\omega_{1\left(p\right)}}{\omega_{2}\left(p\right)}$ is a constant that does not depend on *p.* In other words, the ratios of comparable percentiles always produce the same number when you compare two lognormal distributions having the same scale parameter. 

To illustrate how the concept of relative percentiles is used, suppose that we want to compute the effect size of a dichotomous covariate X ^*^ on a log-normally distributed drug plasma concentration *Y_D_*. Suppose that X ^*^= 1 if the patient belongs to patient subpopulation A, and X ^*^= 0 if the patient belongs to subpopulation B. Let α be the regression coefficient of X ^*^in model (1) (X ^*^is a covariate in vector ***X***, and β^*^ is a component of vector ***β***). Then, after controlling for other covariates and drug metabolic activity, any percentile of the distribution of plasma concentrations in subpopulation A equals e^α *^ times the comparable percentile in subpopulation B, and the quantity

$$E=\left(e^{\beta^{*}}-1\right)\times 100\%$$

measures the size of the effect of the covariate X ^*^ on drug plasma concentrations [7, 8, 11, 26]. Moreover, dose correction factors can be computed directly with the formula $e^{{-\beta }^{*}}$ . Regardless of how dosages are being estimated, a patient's dosage should be multiplied by this factor if the patient’s subpopulation status changes from X ^*^= 0 to X ^*^= 1 [see 7].

The above measure of effect size has been used to assess the clinical importance of drug-drug interactions in clinical environments [7, 8]. For instance, Diaz *et al.* [7] investigated the effect sizes of co-medications on plasma clozapine concentrations. Their study included adult patients with schizophrenia taking different types of co-medications, and also patients not taking co-medications (N=255). The patients provided a total of 415 steady-state trough clozapine concentrations (1 to 15 concentrations per patient). A random intercept linear model of the natural log of clozapine concentrations was fit. The study confirmed that phenobarbital induces clozapine metabolism (E = −28%), and that fluoxetine (E = +42%), fluvoxamine (E = +263%) and paroxetine (E = +30%) inhibit it. Interestingly, in drug-drug interaction clinical studies, the sign of the effect size *E* can be interpreted in terms of metabolism induction (negative sign) or inhibition (positive sign). This study also found that valproic acid inhibits clozapine metabolism in non-smokers (E = +16%). In contrast, valproic acid induces clozapine metabolism in smokers (E = −22%); moreover, after confirming that smoking induces clozapine metabolism, it was computed that this induction may be stronger when the patient is taking valproic acid.

Similarly, Botts *et al.* [8] investigated the effect sizes of some co-medications on plasma olanzapine concentrations. The study included adult patients with schizophrenia taking co-medications, and patients not taking co-medications (N=163). The patients provided a total of 360 olanzapine concentrations (1 to 11 measures per patient), and model (1) was fit. This study found that olanzapine concentrations were 10% lower in non-smokers who were taking lamotrigine than in non-smokers who were not taking lamotrigine, and that olanzapine concentrations were 35% higher in smokers who were taking lamotrigine than in smokers who were not taking lamotrigine. Thus, lamotrigine decreased olanzapine metabolism in smokers, and may increase it slightly in non-smokers. Also, olanzapine concentrations were 41% lower in smokers who were not taking lamotrigine than in non-smokers who were not taking lamotrigine, and olanzapine concentrations were 11% lower in smokers who were taking lamotrigine than in non-smokers who were taking lamotrigine. Thus, lamotrigine comedication may reduce the inducing effects of smoking on olanzapine metabolism. The point is that random effects linear models may provide interpretable measures of the clinical importance of comedications in a clinical environment. In general, measures of effect sizes based on relative percentiles are suitable for quantifying the extent of the effects of different types of clinical, demographic, genetic or environmental covariates on pharmacokinetic or pharmacodynamic responses.

## 5 LINEAR VERSUS NON-LINEAR MODELS

The strong influence of compartmental models in theoretical pharmacology may explain why many pharmacologists tend to belittle the importance of statistical linear models in current and past pharmacological research, despite the well-known fact among pharmacologists that a simple log-transformation of a pharmacological response may facilitate the analysis of pharmacological data, particularly pharmacokinetic data, and despite the fact that regulatory agencies have issued some guidelines for statistical analyses of pharmacological data that rely heavily on log-transformations and random-effects linear models (see, for instance, the United States Food and Drug Administration's guidelines for statistical analysis in bioequivalence studies [28]). Surprisingly, many pharmacologists do not seem to be aware that the reason log-transformations work very well in the statistical analysis of data from many pharmacological studies is that this "mathematical trick" frequently produces pharmacological linear models [see, e.g. 29, 30]. 

Population pharmacokinetic linear mixed models have several advantages over nonlinear mixed models. First, linear mixed models are easier to build and fit to phase III and IV data. In fact, variable selection with nonlinear models is by far more complicated. More importantly, the statistical estimation theory of linear models is much more developed than the theory of nonlinear models, particularly with small to moderate sample sizes; also, the numerical methods used for fitting linear models to data are more reliable and less controversial than the numerical methods used in nonlinear modeling. As a result, p-values testing the significance of covariate effects in mixed-effects linear models are less controversial [9]. It must be emphasized, however, that the problem of deciding which modeling approach should be used is an empirical and not a theoretical or numerical one; that is, it is the data and practical considerations that dictate which model is more appropriate. 

Another factor is that absorption parameters in nonlinear models obtained from compartmental theory are difficult to estimate with data from phase III and IV studies, because the designs in these studies are usually sparse [31]. The problem is that compartmental models always include an absorption parameter when representing the pharmacokinetics of an oral drug. However, to design a dosage regime for a chronically ill patient, absorption parameters are irrelevant [19]. Absorption parameters are not included in model (1), which facilitates its use in the context of phase III and IV studies. Moreover, in the case of a pharmacokinetic response with linear pharmacokinetics, model (1) is essentially a model of the drug steady-state concentration to dosage ratio, whose variations are mainly governed by clearance variations. Clearance, in turn, is the most relevant pharmacokinetic quantity to consider when the goal is dosage individualization and adjustment [19].

## 6 RANDOM-EFFECTS LINEAR MODELS AND EVIDENCE FARMING

A considerable gap between the type of practice that supporters of evidence-based medicine (EBM) advocate and real clinical practice has been pointed out [2]. In fact, clinical practice guidelines written with an EBM approach usually rely on results from studies reporting only population averages, and on studies conducted with exogenous populations; this seems to be at odds with the precepts of personalized medicine which aims at finding optimal treatments for all patients, not only for an average or a non-local patient [32]. 

As an alternative to EBM, a futuristic concept called “evidence farming” (EF) has been proposed whose development will rely heavily on internet technology [2, 3]. In EF, a health care provider enters medical information from an individual patient into a web-site-based system that will help the provider design a treatment regime for the patient. To find an optimal treatment, analytical tools are used that will combine information from the current patient with information from similar patients who have been treated in the past by the same or other providers. Very importantly, patient outcomes are also entered into the system, and all the entered information remains in the system in order to build an increasing body of knowledge that will benefit future patients. The main idea is that the system will help clinicians learn from their own or others’ past experience, and will help make decisions about individual patients [2, 3]. 

In addition to the technological challenges that EF faces, appropriate tools for data analysis and treatment computations will need to be developed and implemented. Statistical mixed models may play an important role in this enterprise. From a purely conceptual point of view, the goals of EF are not much different from those of the empirical Bayesian philosophy: collect as much information from each patient as ethically and economically as possible, and from as many patients as clinicians can, use all this information to build a body of knowledge (the mixed model for the empirical Bayesian) and, when a new patient needs to be treated, information from the patient should be combined with the body of knowledge in order to produce an informed decision about how to treat the patient. However, the empirical Bayesian philosophy additionally prescribes decision rules that are solidly justified through decision-theory principles.

Thus, as can be inferred from the above discussion on Diaz *et al.*’s algorithm, random-effects linear models and empirical Bayesian approaches may provide some of the analytical and computational tools that evidence farming will need to achieve its goal of helping health care providers to tailor treatments to individual patients. 

## 7 RANDOM-EFFECTS LINEAR MODELS AND PHARMACOGENOMICS

Advances in pharmacogenomics have led to genome-wide association studies (GWAS) which have the potential for examining the applicability of millions of genetic variations to personalized medicine. However, the methodology for performing such examination is still underdeveloped and, despite the fact that corrections for multiple comparisons are routinely used in these studies, many of the genes that are identified as significant in these studies are false positives [33]. 

Blindly searching for genetic variants affecting a pharmacological response, that is, searching without the help of carefully stated pharmacological and clinical hypotheses and without considering biological plausibility, is probably one important reason for the large amount of false findings reported by GWAS. Just searching for significant associations between gene variants and a pharmacological response is not enough, and it is possible that examining biological plausibility and discarding associations that are not consistent with pharmacological knowledge [34, 35] may be more fruitful than just correcting for multiple comparisons. Next, we suggest how model (1) can be used to design pharmacogenomic studies that exploit prior pharmacological and clinical knowledge and the structure of this model. 

As mentioned above, it is usually hypothesized that the random intercept α in model (1) incorporates all the variability of the pharmacological response *Y_D _*that is caused by genetic heterogeneity, as well as some inter-individual variability caused by other factors. Moreover, since ε is an intra-patient random error, the variability of ε does not reflect genetic variability across patients. We suggest here that we can make the above hypothesis work in our favor in order to systematically search for genetic variants affecting the pharmacological response, provided that model (1) is used, provided that appropriate environmental, clinical or demographic covariates are measured and included in the model, and provided that pharmacological knowledge allows assuming that these measured non-genetic covariates explain most of the non-genetic variability of the pharmacological response. Under these conditions, a genetic covariate added to the model should substantially reduce the variability of α for it to be considered clinically relevant to the pharmacological response.

For instance, suppose that we want to test whether a particular genetic variant affects clozapine levels by using data from a sample of patients. According to previous studies using model (1), gender, smoking and comedications are probably the most important non-genetic variables affecting clozapine levels [5, 7]. Also, it is reasonable to assume that the variability of the intercept of a random-intercept linear model including the above covariates may be almost totally explained by genetic heterogeneity, since the above covariates may explain almost all non-genetic variation in clozapine levels. Thus, if the genetic variant really affects clozapine levels, and if a covariate constructed with this variant is added to the model, then we must observe two things: 1) the regression coefficient of this genetic covariate should be significant, and 2) the variance of α, *i.e*. $\sigma_{\alpha }^{2}$ , should be significantly reduced. Moreover, if the addition of the genetic covariate to model (1) is associated with a reduction in the variability of ε, then the genetic variant will probably not explain variations in clozapine levels. In other words, if the regression coefficient of the genetic covariate is statistically significant after adjusting for smoking, gender and comedications, but the addition of the genetic covariate to the model did not cause a significant reduction in the variance of α, or the addition caused a reduction in the variance of *ε*, then the statistical significance of the regression coefficient is probably a false positive. 

Thus, in a pharmacogenomic study of clozapine levels using model (1) as the response, a genetic variant should seriously be considered for future studies only if 3 facts are *simultaneously* observed: (1) its regression coefficient is significantly different from 0 when smoking, gender and relevant comedications are also covariates in the model; (2) its addition to the model significantly decreases the variance of α and (3) its addition to the model does not significantly decrease the variance of ε. This approach should produce fewer false positives than just testing the association between the genetic variant and clozapine levels because the potentially confounding effects of non-genetic covariates are controlled for, and because the requirement that the genetic variant satisfies several hypotheses simultaneously reduces the probability of type I error [36].

## 8 CONCLUSIONS AND OUTLOOK

We have examined evidence that suggests that random effects linear models may provide accurate representations of phase III and IV pharmacokinetic data. In particular, there is empirical evidence that linear models with log-transformed drug steady-state concentrations may be useful tools for describing the pharmacokinetic effects of covariates. Although studies exploring the use of these models with pharmacodynamic responses are needed, the applicability of these models to these responses seems to be appropriate and very probably useful and productive. Empirical, theoretical and simulation results suggest a potentially wide applicability of linear mixed models to drug dosage computations and personalized medicine. In particular, these models may provide the computational and conceptual tools that are necessary to implement medical-treatment individualization in web-sites supporting evidence farming. Finally, the special way in which these models separate different sources of pharmacological variability allows using them as tools for designing pharmacogenomic studies, especially when prior knowledge on the environmental factors that affect the pharmacological response of interest is available. 

## Figures and Tables

**Table 1.** Applications of random-effects linear models in personalized medicine.

Dynamic drug dosage individualization through bayesian feedback. Computation of dose correction factors with phase III and IV PK data. Computation of minimum number of blood samples from a patient for finding an optimal individualized dosage in therapeutic drug monitoring. Measuring the clinical importance (effect sizes) of clinical, demographic, environmental or genetic covariates. Study of drug-drug interactions in clinical environments. Computational tools for implementing evidence farming in web sites. Test of the effects of gene variants on PK or PD responses in pharmacogenomic studies. Development of pharmacological theory that provides a definition of optimal individualized drug dosage and mathematical tools for examining the optimality of dosage regimes.

PK: Pharmacokinetic; PD: Pharmacodynamic

## ABBREVIATIONS

CYP: = Cytochrome P450
EBM: = Evidence-based medicine
EF: = Evidence farming
GWAS: = Genome-wide association studies
TDM: = Therapeutic drug monitoring
